# Practicing precision medicine with intelligently integrative clinical and multi-omics data analysis

**DOI:** 10.1186/s40246-020-00287-z

**Published:** 2020-10-02

**Authors:** Zeeshan Ahmed

**Affiliations:** 1 Institute for Health, Health Care Policy and Aging Research, Rutgers University, 112 Paterson Street, New Brunswick, NJ USA; 2 Department of Medicine, Robert Wood Johnson Medical School, Rutgers Biomedical and Health Sciences, 125 Paterson Street, New Brunswick, NJ USA

**Keywords:** Precision medicine, Clinics, Genomics, Metabolomics, Integrative analysis, A﻿rtificial intelligence, Machine learning

## Abstract

Precision medicine aims to empower clinicians to predict the most appropriate course of action for patients with complex diseases like cancer, diabetes, cardiomyopathy, and COVID-19. With a progressive interpretation of the clinical, molecular, and genomic factors at play in diseases, more effective and personalized medical treatments are anticipated for many disorders. Understanding patient’s metabolomics and genetic make-up in conjunction with clinical data will significantly lead to determining predisposition, diagnostic, prognostic, and predictive biomarkers and paths ultimately providing optimal and personalized care for diverse, and targeted chronic and acute diseases. In clinical settings, we need to timely model clinical and multi-omics data to find statistical patterns across millions of features to identify underlying biologic pathways, modifiable risk factors, and actionable information that support early detection and prevention of complex disorders, and development of new therapies for better patient care. It is important to calculate quantitative phenotype measurements, evaluate variants in unique genes and interpret using ACMG guidelines, find frequency of pathogenic and likely pathogenic variants without disease indicators, and observe autosomal recessive carriers with a phenotype manifestation in metabolome. Next, ensuring security to reconcile noise, we need to build and train machine-learning prognostic models to meaningfully process multisource heterogeneous data to identify high-risk rare variants and make medically relevant predictions. The goal, today, is to facilitate implementation of mainstream precision medicine to improve the traditional symptom-driven practice of medicine, and allow earlier interventions using predictive diagnostics and tailoring better-personalized treatments. We strongly recommend automated implementation of cutting-edge technologies, utilizing machine learning (ML) and artificial intelligence (AI) approaches for the multimodal data aggregation, multifactor examination, development of knowledgebase of clinical predictors for decision support, and best strategies for dealing with relevant ethical issues.

## Background

Since the beginning of scientific discoveries, it has been central to understand the cause of disease and senescence [1]. Pain is one of the key triggers for patients to seek diagnosis and treatment. However, when dealing with some of the life-threatening diseases, patients may not feel pain. To identify and help patients with known diseases and symptoms, and those heading toward late stages of novel infectious (e.g., COVID-19), chronic (e.g., diabetes, heart disease), acute (e.g., flu, stroke, heart attack), and complex (e.g., cancer) diseases, it is essential to provide timely personalized treatment [2–10]. Our evolving understanding of the complex nature has led us to realize that to effectively diagnose and treat patients with these conditions, it is essential to provide personalized utilize a precision medicine approach [10]. Progress in the molecular technology developments have led to vast amounts of human health-related data that are expected to greatly expand our understanding of human biology and health, and to drive personalized medicine. We hypothesize that on-demand access and analysis of clinical, genetic, and metabolic data will align biomarker identification with treatment windows necessary for real-time personalized care and enhance prediction of potential disease risks [11]. Despite current advancements, there is still no platform available that can efficiently integrate clinical, multi-omics, and epidemiological data acquisition, and enable effective management of data analytics with a user-friendly physician-oriented clinical interface [12, 13]. Platforms like The Cancer Genome Atlas (TCGA) [14] provide a great resource for scientific data (i.e., genomics or epigenetics sequence data) but offer limited capacity for clinical information, because they are not directly integrated to clinical health systems like Epic, NextGen, and Cerner etc. The inability of disparate platforms to effectively integrate is largely due to the high volume and heterogenous nature of the different types of data they contain, which is acquired from variable sources, each with unique data structures. It is essential to address a major gap in developing precision diagnostics and therapeutic agents in healthcare by establishing a digital solution for practicing precision medicine. Intelligent big data platforms are necessary to improve the quality of care-delivery process by increasing permeation of electronic health record (EHR) systems into clinical environments, focusing on predictive diagnosis, enabling real-time telemedicine, and precise treatment resulting in lower spending on life-threatening complex and chronic diseases [15].

## Practicing precision medicine and AI

**Fig. 1:**
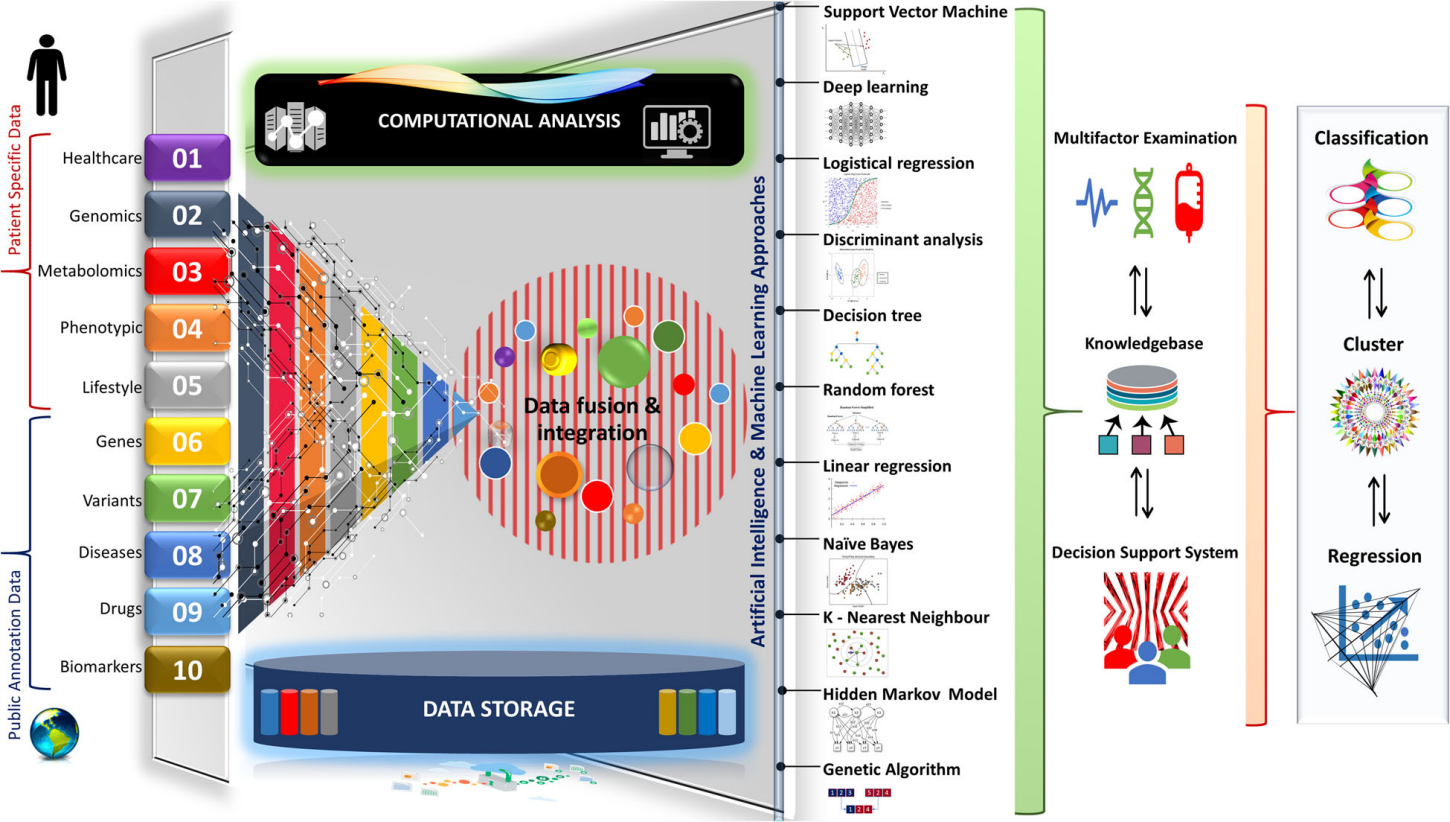
Design modeling of heterogenous patient-specific healthcare, genomics, metabolomics, phenotypic, and lifestyle data, and publicly available annotation data including genes, variants, diseases, drugs, and biomarkers. Analysis using AI and ML approaches (Support Vector Machine, Deep Learning, Logistic Regression, Discrimination Analysis, Decision tree, Random Forest, Linear Regression, Naïve Bayes, K-Nearest Neighbor, Hidden Markov Model, and Genetic Algorithm), multifactor examination, knowledgebase and decision support system for data classification, cluster, and regression analysis. Furthermore, resource allocation for data storage and computational analysis

Precision medicine has the potential to improve the traditional symptom-driven practice of medicine, and allow earlier interventions using predictive diagnostics and tailoring better-personalized treatments [2–9, 16, 17]. However, practicing precision medicine is not straightforward, as significant efforts are required from the experts in multidisciplinary sciences. This necessitates development of progressive healthcare environment that will enable clinicians and researchers to gain a complete picture of the patient to deepen their understanding, using additional details from healthcare and multi-omics data. We hypothesize that clinical information will enrich genomics and metabolomics data such that combined predictors will perform better than the individual classifiers only based on either genomics, metabolomics, or clinical data. Practically supporting the hypothesis, we need to design research methodology, which includes modeling of patient-specific (healthcare, genomics, metabolomics, proteomics, and lifestyle) and publicly available annotation (genes, variants, diseases, drugs, biomarkers) data storage, management, integration, knowledgebase creation, and analysis using different artificial intelligence (AI) and machine learning (ML) approaches (Fig. 1) [18, 19].

Precision medicine is moving forward but with many challenges that require addition of useful analytic tools, technologies, databases, and approaches to efficiently manage massive heterogeneous data, augment networking and interoperability of clinical, laboratory, and public health systems. A major barrier to implementation of precision medicine is the data analysis requirement. Most of the precision medicine efforts today are manual or semi-automated, time-consuming, and unable to facilitate on-demand analysis of diverse human datasets to impact critical treatment windows and predict potential disease risks [19–27]. The traditional way of computational analysis is based on running a series of command-line applications, which require good programming skills and ability to work in the UNIX environment. It hinders linking information generated at different stages of treatments and experiments conducted at levels of sampling, sequencing, and analysis. While precision medicine analyses require complex coordinated efforts between disparate groups with non-aligned data formats and massive amounts of computing time that is essential in many cases to positively impact treatment outcomes. Furthermore, it is difficult but mandatory to address ethical and social issues related to healthcare data collection, privacy, and protection with effective balance [18, 28]. Further, current potential pitfalls are given in attached Table 1.

**Table 1:** Current potential pitfalls

Number	Potential pitfalls
1	Uneven distribution of informatics resources.
2	Integration of biomedical data located among heterogeneous sources.
3	Hazards in dehumanization of healthcare data.
4	Handling of extensively available irrelevant, error prone, and missing data.
5	Intelligent and user-friendly interface development.
6	Applying regulations and policies for data collection, usage and sharing.
7	Harmonizing big data with the definitions of clinical phenotypes and diagnosis.
8	Inflexible EHR database schemas not geared for precision medicine.
9	Lack of data availability on social determinants of health.
10	Unstandardized genomics tools and modifications in their versions and outcome format.
11	Overloaded Data generated during unnecessary follow-up diagnoses and treatments.
12	Augmented computational complexity with increasing number of attributes.
13	Slow SQL based high volume data processing speed.
14	Determining optimal parameters and understanding structures of AI and ML algorithms.
15	Handling continuous explanatory variables with more than two levels and understanding odds and probabilities in AI and ML algorithms.
16	Possibility of too many overfitting attributes in AI and ML algorithms.
17	Handling redundant attributes, distribution of statistically independent attributes, and management of class frequencies affecting accuracy.
18	Reduced evidence and reproducibility.
19	Correct predictor variables selection, and evidence-based observational data analysis and screening.
20	Gaining confidence of clinicians at AI produced results.
21	Ethical and social issues related to healthcare data collection, privacy and protection.

The efficient use of information technology, data science, and AI has the potential to enhance public health surveillance and tracking, with systematic collection, management, analysis, and interpretation of data within accelerated timelines [6, 19, 29–31]. We need detailed bioinformatics and AI platforms for supporting real-time processes involved in multisource heterogeneous raw data generation, mathematical modeling, computational analysis, data fusion, integration, management, and visualization (Fig. 1). Platforms need to be user-friendly, multi-functional, and multi-roles-based to address complex and big data-oriented problems in clinical settings. It will support categorizing interaction patterns among variables, learning from experiences, and strategizing and predicting better orientations. Multiple AI and ML algorithms (e.g., Support Vector Machine, Deep Learning, Logistic Regression, Discrimination Analysis, Decision tree, Random Forest, Linear Regression, Naïve Bayes, K-Nearest Neighbor, Hidden Markov Model, and Genetic Algorithm, etc.) are available for multifactor examination, scientific knowledge extraction, and decision support system (Fig. 1) [32–36]. However, determining which AI and ML approaches to use for which task is a challenge in itself [37]. We suggest classifying tasks based on the available predictor variables, as a key to correctly address this problem. Best fitting use of ML and AI algorithms have the potential to predict the existence of life-threatening diseases risk susceptibility, starting from the most common to rare among the population data [19].

AI has the ability to improve identification of relevant variables for patient data stratification with timely detection of statistical patterns across millions of features to identify conditions that are likely to manifest later and discover modifiable risk factors that support the best utilization of known therapies [38]. Impactful and automated implementation of AI and ML can elevate investigating correlation and overlapping of reported diagnoses of a patient in clinical data, and assess genotype and phenotype associations among various diseases to find potential indistinct results for patient care from highly expressed genes and disease-causing variants [9, 39]. Understanding how genetic variations contribute to health is one important aspect of precision medicine, where additional approaches involve measuring levels of proteins and metabolic products. By harnessing the power of metabolomics, we need to profile a patient’s metabolome and correlate it with their body mass index (BMI). Further, AI can assist in finding metabolite penetrance using listed features and abnormalities, and analyzing biochemical pathways in metabolites [40–42] with patterns of multimodal distributions for candidate genes [10, 43].

## Conclusions

The scientific approach would be to perform analysis of individual genomes giving rise to a new form of preventive and personalized medicine in healthcare. Availability of gene-based designer drugs, precise targeting of molecular fingerprints for tumor, appropriate drug therapy, predicting individual susceptibility to disease, diagnosis, and treatment of mental illness are all a few of the many transformations expected in the decade to come. Precision medicine will timely enable clinicians to integrate healthcare data with targeted assays and tests to identify and assess disease biomarkers and risks, determine actionable genetic variants in patients, obtain the entire picture of the metabolome, and map metabolites to disease pathways.

## Data Availability

Not applicable.

## Abbreviations

AI: Artificial intelligence
BMI: Body mass index
COVID-19: Coronavirus disease 2019
EHR: Electronic health record
HIPAA: Health Insurance Portability and Accountability Act
ML: Machine learning
TCGA: The Cancer Genome Atlas
